# Hypokalemia, hypomagnesemia, hypocalciuria, and recurrent tetany: Gitelman syndrome in a Chinese pedigree and literature review

**DOI:** 10.1002/ccr3.874

**Published:** 2017-03-17

**Authors:** Ming‐Feng Xia, Hua Bian, Hong Liu, Hui‐Juan Wu, Zhi‐Gang Zhang, Zhi‐Qiang Lu, Xin Gao

**Affiliations:** ^1^ Department of Endocrinology and MetabolismZhongshan HospitalFudan UniversityShanghaiChina; ^2^ Department of NephrologyZhongshan HospitalFudan UniversityShanghaiChina; ^3^ Department of PathologySchool of Basic Medical SciencesFudan UniversityShanghaiChina

**Keywords:** Gitelman syndrome, phenotype–genotype relation, review, SLC12A3

## Abstract

Gitelman syndrome is an autosomal recessive disease mostly associated with loss‐of‐function mutations of the *SLC12A3* gene and featured by clinical hypokalemia, hypomagnesemia, hypocalciuria, and histologically hypertrophy of the juxtaglomerular apparatus. A novel homozygous mutation (p.Arg399Pro) at the extracellular domain of SLC12A3 was found and correlated with the severe clinical manifestations.

## Introduction

Gitelman syndrome (GS; Mendelian Inheritance in Man 263800) is an autosomal recessive inherited disease mostly associated with loss‐of‐function mutations of the *SLC12A3* (solute carrier family 12 member 3) gene 1, which encoded the thiazide‐sensitive sodium chloride co‐transporter (NCCT) of the distal convoluted tubule (DCT) 2. The prevalence of GS is approximately 1% as heterozygotes and 1 in 40,000 as homozygotes 2. Clinical symptoms for this disease are hypokalemia, hypomagnesemia, hypocalciuria, and hypochloremic metabolic alkalosis 3. At present, hypokalemia is a common clinical disorder in approximately 20% of hospital inpatients 4, and GS must be considered as a differential diagnosis of some settings of clinical hypokalemia.

The pathogenic gene of GS, *SLC12A3* gene (GeneID: 6559; MIM: 600968; GeneBank: NC_000016.10) is located on human chromosome 16q13 and consists of 26 separate exons 5. There have been over 425 *SLC12A3* mutations found to be associated with GS 6, 7 using human genome database search (HGMD, http://www.hgmd.org) and may show a considerable phenotypical variability clinically 8. Most patients with GS were asymptomatic or mild 9, but a few may show an early onset, severe neuromuscular manifestations (e.g., tetany, seizures, and rhabdomyolysis) 10. The potential explanations for the phenotypical variability in GS may include genetic heterogeneity, position, and nature of mutations 11. However, the correlation between the type and position of *SLC12A3* gene mutation and the severity of clinical symptoms in GS has not been fully deciphered yet. Therefore, identification of novel mutation and its associated clinical manifestations will provide further insights into the functional domains of NCCT and the genotype–phenotype correlations in GS patients, which may lead to personalized treatment.

In this study, we report a Chinese patient with a homozygous p.Arg399Pro mutation in the *SLC12A3* gene, who had severe clinical manifestations and typical pathological features of GS. We also review the literature, provide an outline of *SLC12A3* mutations in Chinese population, and analyze the associations of *SLC12A3* mutation types and sites with clinical manifestations.

## Case Report

### Clinical data of proband and other family members

A 29‐year‐old Chinese woman was referred to our Endocrinology unit with a 10‐year history of fatigue, recurrent tetany, and aggravation of these symptoms for 1 h. She once visited the local hospital and was found to have hypokalemia, but further diagnosis was not made for the cause of hypokalemia. She felt better after supplementation of potassium chloride, but still experienced recurrence and aggravation of symptoms after that. She had frequent cramps on her face, and physical examination revealed a sitting blood pressure of 110/70 mmHg. She did not have a history of long‐term use of laxatives, diuretics, ethanol, or drug addiction nor a history of hypertension. Her parents were nonconsanguineous, and her family members were all asymptomatic.

### Laboratory findings

The laboratory findings revealed severe hypokalemia (1.9 mmol/L, reference: 3.5–5.3 mmol/L), hypomagnesemia (0.45 mmol/L, reference: 0.67–1.04 mmol/L), metabolic alkalosis (PH 7.55 and plasma bicarbonate 36 mmol/L, reference: 23–29 mmol/L), hyperkaluria (57.7 mmol/24 h), and hypocalciuria (urine calcium/creatinine ratio = 0.023, reference: 0.05–0.57 mmol/mmolCRE). The plasma aldosterone (216 pg/mL, reference: 59.5–173.9 pg/mL) and renin activity (2.4 ng/mL/h, reference: 0.05–0.79 ng/mL/h) were increased, and the creatine kinase was extremely high (25,355 U/L, reference: 34–174 U/L) on admission.

### Genotype analysis

**Figure 1:**
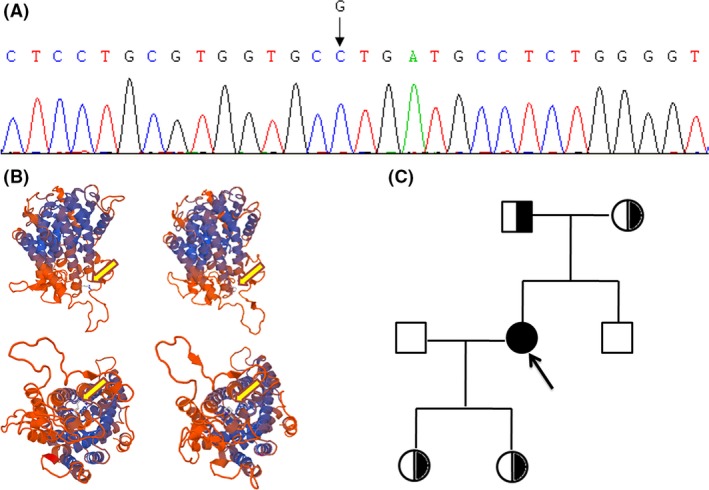
Germline mutation in the pedigree. (A) Electropherogram of the proband revealing a homozygous c.G1196C point mutation. (B) Prediction of three‐dimensional structure of the wild‐type (left) and mutant (right) SLC12A3 protein. Yellow arrows show substitution of the hydrophilic amino acid arginine at codon 399 by a hydrophobic amino acid proline at the extracellular region of SLC12A3 protein. (C) Pedigree of the GS family.

The patients and her family members were screened for possible mutations associated with urinary diseases, including *SLC12A3* and *CLCNKB* genes, by next‐generation sequencing (NGS). Sanger sequencing was used to confirm the detected mutation in the proband. The details of mutation analysis were shown in Appendix S1. Sequence analysis of the *SLC12A3* gene revealed a homozygous mutation in exon10 (c.1196G>C) (Fig. 1A). The molecular structures of the normal and mutant SLC12A3 proteins were modeled using the SWISS‐MODEL protein structure modeling server (www.swissmodel.expassy.org) 12. This change is predicted to substitute the hydrophilic amino acid arginine at codon 399 by a hydrophobic amino acid proline, which is located at the extracellular region of NCCT protein. The predicted molecular structure of the wild‐type SLC12A3 and p.R399P protein was shown in Figure 1B. The mutation was also found at a single heterozygous state in her parents and two daughters (Fig. 1C).

### Renal histological examination

**Figure 2:**
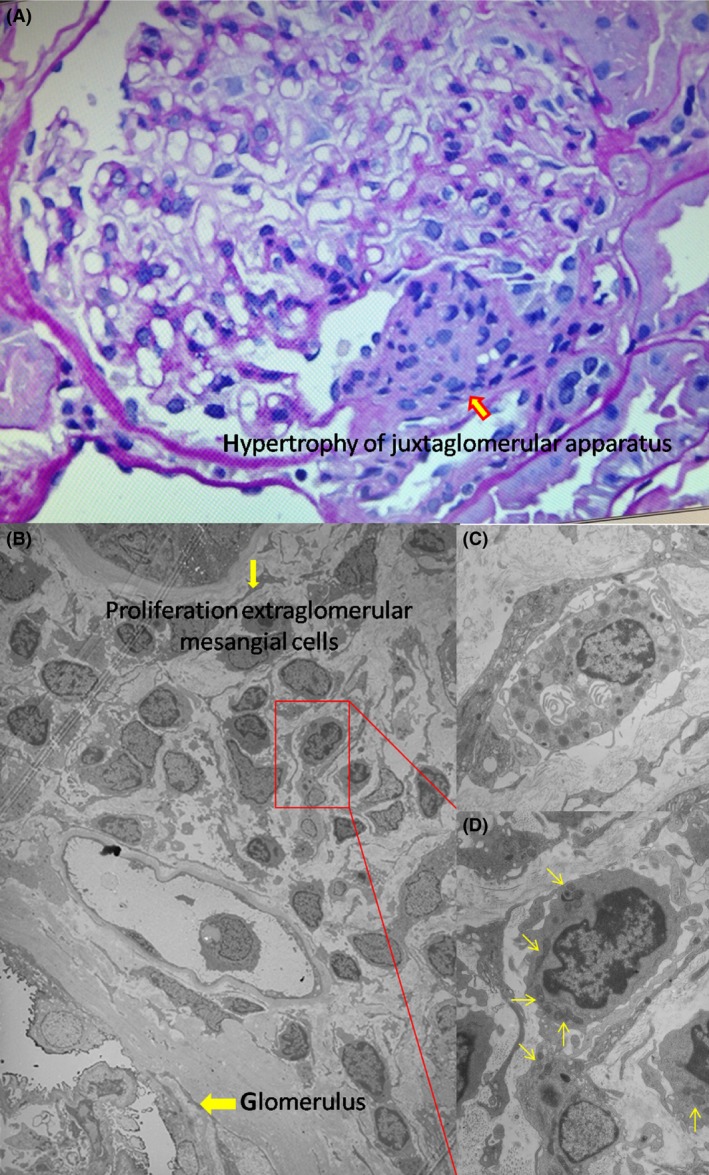
Renal biopsy of the case presenting with Gitelman syndrome. (A) Light microscopic examination showed hypertrophy of juxtaglomerular apparatus (more than 50% of glomeruli involved) with proliferation of extraglomerular mesangial cell. The morphology of glomeruli is almost normal. (B) Electron microscopy revealed proliferation of extraglomerular mesangial cells. (C, D) Numerous secretive granules in the cytoplasm of this extraglomerular mesangial cell.

To determine the effect of SLC12A3 p.R399P mutation on the kidney histology, a renal biopsy was performed 10 days after correction of serum creatine kinase level (Fig. 2). Renal tissue was obtained through percutaneous needle biopsy performed at department of nephrology, Zhongshan Hospital (Shanghai, China). The tissue sample was processed for light microscopy and electron microscopy examinations according to the standard protocols of Department of Pathology, School of Basic Medical Sciences, Fudan University (Shanghai, China). Light microscopy showed hypertrophy of the juxtaglomerular apparatus with proliferation of extraglomerular mesangial cells with normal morphology of glomeruli (Fig. 2A). Proliferation of extraglomerular mesangial cells was also found under electron microscopy (Fig. 2B), and there were many secretive granules in the cytoplasm of extraglomerular mesangial cells (Fig. 2C and D).

### Treatment and follow‐up

After the clinical diagnosis was confirmed, the patient received the following treatment: potassium chloride sustained release tablets 1 g, potassium–magnesium aspartate oral liquid 10 mL and spironolactone 80 mg, taken orally three times daily. After the treatment, the symptoms improved, the serum potassium and magnesium increased to the range of 3.2–3.5 mmol/L and 0.63–0.83 mmol/L, and serum creatine kinase level recovered within the normal range. Severe hypokalemia has not recurred during a one‐year follow‐up after discharge.

### Correlation between phenotype and genotype

**Figure 3:**
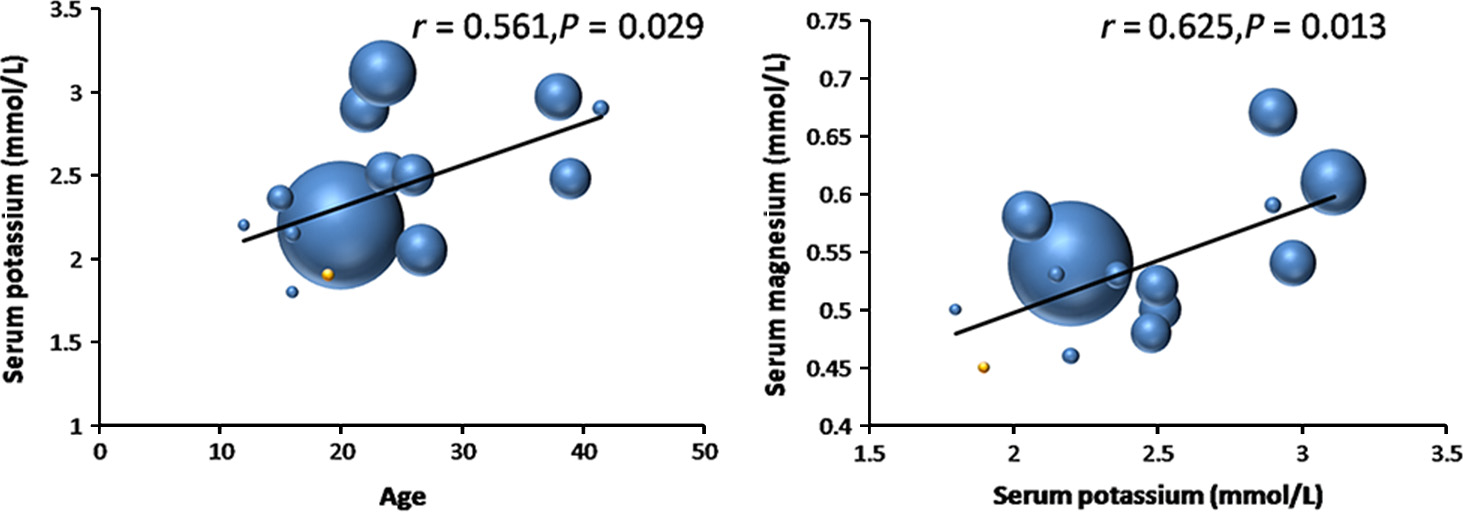
Associations of serum potassium with age at onset (panel on the left) and serum magnesium (panel on the right). The sizes of bubbles represent the sample sizes of different studies.

To find the possible explanation for the severe clinical manifestations of the GS patient in our current study, the data of all Chinese GS patients in previous publications, which could be found in the PubMed database to date, were collected to analyze the associations between the types and sites of *SLC12A3* gene mutations and severity of clinical manifestations in GS. As shown in Table 1, 252 patients (157 male and 95 female) with GS have been described in the literature 6, 13, 14, 15, 16, 17, 18, 19, 20, 21, 22, 23, 24, 25. The average age at onset was 23.6, and hypomagnesemia and hypocalciuria were found in 92% and 88% of GS patients (Table 1). Patients with early onset of symptoms had significantly lower serum potassium level (*r* = 0.561, *P* = 0.029), and the serum magnesium level was positively associated with serum potassium level (*r* = 0.625, *P* = 0.013) by Pearson's correlation analyses (Fig. 3). The mutations were distributed throughout the whole *SLC12A3* gene. To analyze the correlation between the types and sites of *SLC12A3* mutations and clinical manifestations, we divided the patients of GS into groups of single heterozygotes, complex heterozygotes, and homozygotes, and the mutated alleles were classified as intracellular, transmembranal, and extracellular mutations according to their sites. Complex heterozygotes/homozygotes of *SLC12A3* gene mutations had significantly lower serum potassium than the single heterozygotes, and the patients with *SLC12A3* mutations extracellularly also had significantly lower serum potassium and magnesium levels than those with mutations transmembranally or intracellularly (Table 2).

**Table 1:** Summary of Gitelman syndrome with mutation information in Chinese

Author, year	Location	Mutation	Consequence	Male/Female	Age at onset	K^+^, mmol/L	Mg^2+^, mmol/L	Hypocalciuria n(+)/n(−)	Hypomagnesemia n(+)/n(−)
Zha (2015) 13	SH	c.G791C	p.G264A	0/1	12	2.2	0.53	1/0	1/0
Luo (2015) 6	FJ	c.C2782T, c.C2129A	p.R928C, p. S710X	2/0	41.5	2.9	0.59	2/0	1/1
Lu (2015) 14	SC	c.C488T	p.T163M	1/0	16	1.8	0.5	1/0	1/0
Li (2015) 15	SD	c.C179T, c.234delG, c.G1925A c.486‐490DelTACGGinsA	p.T60M, p.R642H, p.162frameshift	2/0	24.5	2.6	0.46	1/1	2/0
Jiang (2015) 16	BJ	Unknown	p.G439S, p.S615L, p.R399C, p.D486N, p.W151T, p.A370P, p.G800R, p.Q131K, p.G201D, p.V169I, p. L170Q, p.Y70C, p.R861C, p.L215P, p.W844T, p.809Frameshift, p.R913Q, p.V677M, p.S976F, p.T60M, p.L700V, p.T428I, p.G196V, p.959frameshift	14/3	22	2.9	0.67	11/6	12/5
Jiang (2014) 17	BJ	Unknown	p.R655H, p.T60M, p.N566L, p.R913Q, p.556Frameshift.	23/9	23.5	3.11	0.61	Unknown	25/7
Ren (2013) 18	SH	c.C185T, c.T1294G, c.G1322T, c.346‐353delACTGATGG, c.T1718G, c.2969insGCT, c.C2761T, c.C1083G, c.G1322T, c.G2717A, c.C2129A, c.A1163G, Del n7426–n7438, Ins (accgaaaatttt), c.T1639C, c.G1462A, c.G2404T	p.T60M, p.C430G, p.114Frameshift, p.G439V, p.959frameshift, p.L571P, p.997insC, p.R928C, p.N359L, p.R913Q, splice mutation, p.S710X, p.R919C, p.Y386C, p.F545L, p.G800W	7/9	38	2.97	0.54	14/2	16/0
Tseng (2012) 19	TW	Unknown	p.A13P, p.D62G, P.T60M, p.R83Q, p.H90Y, p.R145C, p.T163M, p.L215P, p.H234Q, p.S283Y, p.frameshift, p.T304M, p.R334W, p.N426K, p.N442K, p.S614L, p.N640S, p.R642H/C,p.T649M, p.S710X, p.D848N, p.W844X, p.L858H, p.R871H/S, p.L892P, p.R896X, p.R913Q, p.P947S	70/47	20	2.2	0.54	110/7	109/8
Sung (2011) 20	TW	Unknown	p. T163M, p.T649M, p.688frameshift	1/1	16	2.15	0.53	1/1	2/0
Lo (2011) 21	TW	Unknown	p.frameshift, p.L215P, p.R83Q, p. T163M, p.T60M, p.R871H, p.W844X, p.R642C	12/7	26.7	2.05	0.58	Unknown	12/7
Qin (2009) 22	SH	c.C185T, c.C2761T, c.G1462A, 492_496delTACGGinsA, c.C1022T, c.C1083G,c.G1322T, c.G2717A, IVS7‐1 G > A, g.7427_7438delinsCCGAAAATTTT, c.G2717A, IVS16‐2 A > G, c.G1268T, c.G1970A, c.A1163G	p.T60M, p.R919C, p.D486N,p.162frameshift, p.T339I, p.N359L, p.G439V, p.R904Q, splice mutation, p.C421F, p.R655H,p.Y386C	7/6	23.8	2.51	0.50	Unknown	Unknown
Miao (2009) 23	SD	Unknown	p.T60M, p.T304M, p.T465P, p.N611T, p.C146F, p.N359D, p.T465P, p.P556L, p.N611T, p.Y857C	7/5	39	2.48	0.48	Unknown	Unknown
Shao (2008) 24	SH	c.G593T, c.G1322T, c.C185T, c.T1294G, c.1384delG, c.2969insGCT, c.G1462A, c. 2883‐2884delAG c.346‐353delACTGATGG	p.G196V, p.G439V, p.T60M. p.C430G, p.114frameshift, p.460frameshift, p.959frameshift, p.L571P, p.997insC, p.D486N	9/4	26	2.5	0.52	12/1	12/1
Lin (2004) 25	TW	c.C2135A, c. 2881‐2882delAG	p.S710X, p.959frameshift	2/3	15	2.36	0.53	3/2	5/0

SH1, Shanghai; FJ, Fujian; SC, Sichuan; SD, Shandong; BJ, Beijing; TW, Taiwan.

**Table 2:** Biochemical parameters of GS patients with different types and sites of *SLC12A3* gene mutations

	*SLC12A3* gene mutation type			*P* for trend	*SLC12A3* gene mutation site^a^			*P* for trend
	Single heterozygotes	Complex heterozygotes	Homozygotes		Intracellular mutations	Transmembranal mutations	Extracellular mutations	
Number	13	58	28		57	7	7	
Age	27 (19–34)	25 (17–37)	32 (21–39)	0.177	27 (18–37)	20 (15–37)	28 (19–34)	0.679
Serum potassium, mmol/L	2.75 ± 0.33	2.34 ± 0.46^b^	2.28 ± 0.52^b^	0.029	2.37 ± 0.47	2.32 ± 0.55	2.07 ± 0.16^c^	0.275
Serum magnesium, mmol/L	0.55 ± 0.11	0.56 ± 0.15	0.61 ± 0.18	0.183	0.59 ± 0.14	0.57 ± 0.04	0.44 ± 0.08^c^^,^ d	0.021

a A total of 71 patients with homozygous mutations or complex heterozygous mutations at the same side of cellular membrane were enrolled.

b *P* < 0.05, compared with GS patients with a single heterozygous *SLC12A3* mutation.

c *P* < 0.05, compared with GS patients with *SLC12A3* mutations intracellularly.

d *P* < 0.05, compared with GS patients with *SLC12A3* mutations transmembranally.

## Discussion

Gitelman et al. described the clinical features of GS for the first time in 1966 1. In 1996, Simon et al. cloned cDNA of *SLC12A3* and indentified *SLC12A3* gene mutation as the cause of GS 2. NCCT is coded by *SLC12A3* gene and constituted by 1021 amino acid residue, which predict 12 membrane spanning domain and longer amino and carboxyl end within the cells. In our current study, we reported a GS patient with a novel homozygous mutation in the *SLC12A3* gene (c.1196G>C) that subsequently led to substitution of the basic amino acid arginine with a hydrophobic amino acid proline at the extracellular region of NCCT protein. Phenotypically, this patient had severe GS symptoms of hypokalemia, hypomagnesemia, hypocalciuria, and recurrent onsets of tetany. We also found that the severe clinical manifestations of our patients might be associated with its homozygous mutation at the NCCT extracellular region by analyzing the relationships between clinical manifestations and *SLC12A3* mutation types and sites in all published studies of GS patients in China.

Sodium chloride co‐transporter participates in the control of ion homeostasis at the distal convoluted tubule portion of the nephron. Loss‐of‐function mutations in NCCT will impede the reabsorption of sodium in the DCT, and result in more sodium arriving at the collecting duct and mild volume contraction. To maintain the salt homeostasis, the exchange between Na^+^/K^+^, H^+^/K^+^, and Na^+^/H^+^ was increased in the cortical collecting duct at the expense of increased secretion of potassium and hydrogen ions, which led to hypokalemia and metabolic alkalosis. The low volume also activates the renin–angiotensin–aldosterone system, which stimulates the proliferation of juxtaglomerular apparatus and increases the renin activity and aldosterone levels in GS. On the other hand, the passive Ca^2+^ reabsorption in the proximal tubule and reduced abundance of the Mg^2+^ channel TRPM6 in the DCT explains hypocalciuria and hypomagnesemia, respectively 26.

Pathologically, the patient was characterized by hypertrophy of the juxtaglomerular apparatus, and we also found many secretive granules in the cytoplasm of extraglomerular mesangial cells under electron microscopy, which conformed to the typical pathologic manifestations of GS. Although hypokalemia‐induced rhabdomyolysis was suspected in our patient, her serum creatine level was normal and no renal tubular necrosis was found under light microscope 10 days after correction of serum creatine kinase level. Therefore, it was not likely that the renal pathological change was affected by the rhabdomyolysis.

To date, more than 100 mutations have been reported in Chinese GS patients (Table 1). All patients had hypokalemia and 92% had hypomagnesemia. Our analysis based on the previous reports showed that GS patients with lower potassium were more symptomatic, and usually had a younger age at diagnosis and lower serum magnesium level. It has been reported the co‐localization of NCCT and TRPM6 proteins 17, which might indicate the functional status of NCCT might regulate the Mg^2+^ channel TRPM6.

Traditionally, the GS is recessively inherited, with simple heterozygous relatives being asymptomatic. However, there is still a proportion (13.1%) of affected individuals with only one *SLC12A3* mutant allele detectable in the Chinese GS patients. Single heterozygotes of patients with GS have been reported previously 27. Although a second mutation in some nonstudied region or in other genes cannot be excluded, the patients with *SLC12A3* single heterozygous mutation showed a milder manifestation of hypokalemia in comparison with the complex heterozygotes or homozygotes in the current study (Table 2). It has been reported that one heterozygous mutation in *SLC12A3* gene would partially impair the renal function for salt handing 28. Also, the expression of NCCT may be influenced by epigenetic modifications and/or silent polymorphisms, which lead to impaired function in simple heterozygotes 29. Therefore, it is still necessary to screen for potential hypokalemia in the subjects carrying single heterozygous *SLC12A3* gene mutations.

Previous studies have shown that the *SLC12A3* mutations scattered through the whole coding sequence of the NCCT protein, but most of the mutations are frequently found in the intracellular domains of the protein 30, and the phenotype of GS is highly heterogeneous 11. Jiang et al. found the percentage of mutated alleles distributed extracellularly was greater in hypo‐ than normo‐magnesemic patients 17. In our review of Chinese GS, most of the SLC12A3 mutation alleles were located on the intracellular domains of NCCT, and we found the average serum potassium and magnesium was significantly lower in subjects with SLC12A3 mutations extracelluarly than those with mutations intracellularly or transmembranally (Table 2). Different domains of the NCCT protein have been found to have different functions 31. In general, the basic structure of the Na^+^‐coupled chloride cotransporters features a central hydrophobic domain containing 12 α‐helices that is flanked by a short hydrophilic amino‐terminal domain and a long predominantly hydrophilic carboxy‐terminal domain within the cell 32. There is a long hydrophilic loop connecting transmembrane segments 7 and 8, exhibiting three putative N‐glycosylation sites in extracellular domain of NCCT, which is distinguished with other electroneutral cation‐chloride cotransporters, like the K^+^‐coupled chloride cotransporters (Fig. S1) 29. Interestingly, the p.R399P SLC12A3 mutation of the proband in our study was located at one end of the extracellular long hydrophilic loop of NCCT, and one previous functional study in Xenopus oocytes had shown that NCCT with R399C mutants almost lost the whole function of Na^+^ uptake 29. Therefore, the severe clinical manifestation of the patient in our current study might be related to its mutation site at the extracellular long hydrophilic loop of NCCT.

Gitelman syndrome is a rare inherited disease, and genetic diagnosis is not commonly used in clinical practice, so it is difficult to enroll sufficient GS patients for phenotype–genotype correlation analysis in one single clinical center. Therefore, we collected the data of all GS patients in previous publications and limited the study population to Chinese to avoid the interference of ethnicity. The study is limited for the possible measurement error among different hospitals, although the reference range for serum potassium and magnesium was very similar in different hospitals.

In conclusion, we report a Chinese patient of GS disease with severe clinical manifestations of recurrent tetany. Genetic analysis identifies a novel link between p.R399P mutation in NCCT and GS symptoms, and its homozygous mutation type and mutation site at the extracellular domain of NCCT may correlate with the severe clinical manifestations based on the literature review on the associations between GS manifestations and SLC12A3 mutations in Chinese.

## Authorship

MX, HB, ZL, and XG: designed the whole study. XG and ZL: diagnosed the patient with GS. MX: collected the information of the patient and her family and contacted the patient for routine follow‐up. HL: carried out the kidney biopsy and made pathological diagnosis under light microscopy. HW and ZZ: carried out the electron microscopy examinations. MX and HB: wrote the article and made the literature review.

## Conflict of Interest

All authors state that they have no conflict of interests.

## Supporting information


**Appendix S1.** Methods of genotype analysis.

**Figure S1**. Location of p.R399P SLC12A3 mutation at the extracellular long hydrophilic loop of NCCT.
