# Supplementary material for: Hypokalemia, hypomagnesemia, hypocalciuria, and recurrent tetany: Gitelman syndrome in a Chinese pedigree and literature review
Source: Clin Case Rep. 2017 Mar 17;5(5):578–86. doi: 10.1002/ccr3.874 (PMC5412754; doi:10.1002/ccr3.874)
Supplement: Supplementary file 1 — Appendix S1. Methods of genotype analysis. [file CCR3-5-578-s001.docx]

**Mutation analysis**

Next-generation sequencing (NGS) and DNA sequence analysis

Genomic DNA from peripheral blood leukocytes of the patients and her family members was isolated using BloodGen Mid kit (CWBIO, China) and following the standard procedure. The DNA sample was sheared by sonication. The sheared genomic DNA was then hybridized with a NimbleGen probe capture array. The array covered about 209 genes from OMIM database（http://www.omim.org）which are associated with urinary system, including *SLC12A3* and *CLCNKB* genes. The libraries were first tested for enrichment by qPCR and for size distribution and concentration using the Agilent Bioanalyzer 2100. The samples were then sequenced on an Illumina Hiseq2500. Two parallel reactions were done for each sample.

Data filtering, mapping and variant detection

Raw image files were processed by the BclToFastq（Illumina）for base calling and generating the raw data. The low-quality variations were filtered out using the quality score≥20 (Q20). The sequencing reads were aligned to the NCBI human reference genome (hg19) using BWA. Samtools and Pindel were used to analyzed SNP and indel of the sequence.

Mutation Confirmed by Sanger-Method Sequencing

Sanger sequencing was used to confirm the mutation in *SLC12A3* of proband. The PCR primers and length of PCR product are as follows. Forward primer, 5’-TCTGCGCCTCTGTAAAATGGCAACGACA-3’, Reverse primer, 5’-CACCCCTCAAGACTTAGCCTCTTAGTGC-3’. PCR product was sequenced by ABI 3730XL, and the resulting sequence was compared to the corresponding wild-type sequences of *SLC12A3* (NM_000339.2) by DNASTAR software.
