# Supplementary figures and images for: Hypokalemia, hypomagnesemia, hypocalciuria, and recurrent tetany: Gitelman syndrome in a Chinese pedigree and literature review
Source: Clin Case Rep. 2017 Mar 17;5(5):578–86. doi: 10.1002/ccr3.874 (PMC5412754; doi:10.1002/ccr3.874)

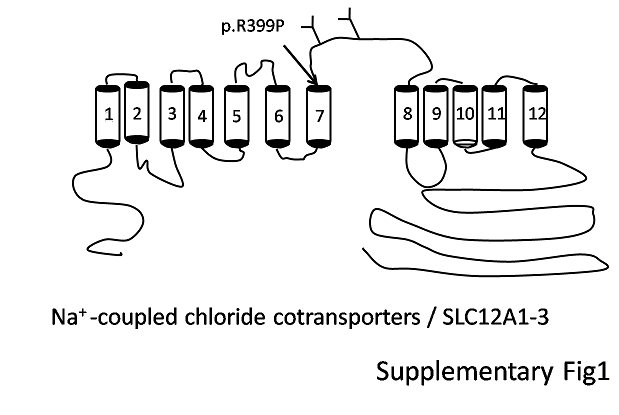

Supplement: Supplementary file 2 — Figure S1. Location of p.R399P SLC12A3 mutation at the extracellular long hydrophilic loop of NCCT. [file CCR3-5-578-s002.tif]
